# Update on Contact Lens Treatment of Keratoconus

**DOI:** 10.4274/tjo.galenos.2020.70481

**Published:** 2020-08-26

**Authors:** Tomris Şengör, Sevda Aydın Kurna

**Affiliations:** 1 Private Practice, İstanbul, Turkey; 2 University of Health Sciences Turkey, Fatih Sultan Mehmet Training and Research Hospital, Clinic of Ophthalmology, İstanbul, Turkey

**Keywords:** Keratoconus, contact lenses, rigid gas-permeable lenses, soft lenses, piggyback lenses, hybrid lenses, scleral lenses

## Abstract

Keratoconus (KC) is a progressive disease that leads to major losses of visual quality and related quality of life. Contact lens (CL) application has a primary place and importance in the correction of the optic problems due to the disease. The corneal changes and increased irregular astigmatism that occur with KC progression necessitate special CL designs and fitting methods. In addition to disease stage, the patient’s lens tolerance also plays a role in the application of CLs in KC patients. With recent advances in materials and design technology, the CLs used in the treatment of KC have developed considerably and there are various types available. In this review, we discuss the wide range of CLs, including rigid and soft lenses, hybrid and scleral lenses, and even custom lens designs, in light of recent scientific advances.

## Introduction

Although contact lens (CL) fitting in keratoconus (KC) is a long and complicated process, it can remarkably improve patients’ quality of life, and therefore should be carried out with patience and care. In other words, every well-informed and dedicated effort to apply CLs in patients with KC may be a door to illumination or a magic touch that shapes the future of these individuals, who live in a blurry and somewhat hopeless world.

KC is a progressive, non-inflammatory corneal disease characterized by thinning of the central and often the paracentral inferior cornea, steepening of the corneal curvature, and asymmetry between superior and inferior keratometric values, resulting in irregular myopic astigmatism. Corneal changes are usually bilateral but asymmetric. Unilateral KC has been described at rates of 0.5-4.5%, but with current topography and corneal aberration measurement techniques, it has been reported that changes that may be associated with subclinical KC can also be detected in the apparently normal fellow eye.^1^ Age of onset is in the early teens or twenties, with progression continuing until the third or fourth decade, and the rate of progression varies among individuals.^2^ KC is classified as mild, moderate, or severe based on ocular signs and symptoms. In the late stages of the disease, corneal thinning and protrusion increase, higher-order aberrations also increase, and visual acuity deteriorates substantially.^3^

Because the prevalence of KC is affected by many factors including geographic location and the diagnostic criteria used, different rates have been reported around the world.^4,5^ Recent studies indicate that with the use of common diagnostic criteria and advanced technology such as corneal topography, the annual incidence and prevalence of KC may be up to 5-10 times the previous values.^6^ KC affects both sexes. While previous studies indicated no marked difference between the sexes, more recent studies have revealed that KC is more common among males.^6,7^

Family history is present in 10-20% of patients, and there are studies suggesting that genetic inheritance plays an important role.^8^ KC is often concomitant with atopy, vernal keratoconjunctivitis, asthma, sleep apnea, mitral valve prolapse, retinitis pigmentosa, Down syndrome, certain noninflammatory connective tissue diseases, and also rigid CL use and eye rubbing, due to their environmental and mechanical effects.^7,8,9,10^

Although it is usually difficult to establish causal relationships, some of the conditions associated with KC point to genetic factors, while others indicate recurrent mechanical trauma.^9^ Thus, the point of consensus is that KC has a multifactorial and multigenetic nature and that environmental factors also play an important role in the development of the disease, or in other words, that the disease develops due to genetic predisposition together with these environmental effects.^10^ Recent studies have suggested as a possible pathogenic mechanism that cytokines produced in response to epithelial trauma induced by eye rubbing may reduce the durability of the corneal tissue, leading to structural changes that manifest with cone formation.^10^ On the other hand, it has been demonstrated in several studies that redox imbalance due to low levels of antioxidant enzymes in the cornea causes increased oxidative stress in keratoconic eyes, and it is now being suggested that tissue destruction caused by the resulting reactive oxygen/nitrogen species may contribute to stromal thinning in KC.^11^

Each of these facets should be kept in mind when planning treatment, preparing a patient for a lifetime with this chronic disease, and implementing and monitoring optic correction systems, especially CLs.

Recent advances in anterior segment imaging systems have contributed significantly to the early diagnosis and treatment of KC and to the monitoring of pathological changes that occur in KC. Today, advanced computerized corneal topography and tomography systems allow detailed assessment of changes in the anterior and posterior corneal surfaces in KC and changes in corneal thickness that occur as the disease progresses. Furthermore, changes in corneal epithelial thickness are detected with optical coherence tomography, while changes at the cellular level are detected by *in vivo* confocal microscopy, which enables the follow-up of the natural course of this progressive disease as well as facilitates the guidance, supervision, and close monitoring of treatment response in CL applications.^12^

There is no definitive treatment for KC. However, corneal collagen crosslinking can be performed to alter corneal biomechanical properties and stop or slow the progression of ectasia. Nevertheless, CLs are still necessary to improve vision quality before and after these disease-stabilizing procedures.^10^

Because KC is a lifelong disease with a progressive course marked by a gradual decline in the quality of vision, it also seriously impairs quality of life in affected individuals. Survey studies based on the National Eye Institute Visual Function Questionnaire (NEI-VFQ) indicate vision-related impairment of quality of life of patients with KC. However, it was also reported that patients who use CLs have the highest quality of life scores.^13,14,15^

The process of increasing vision quality in KC patients is usually complex and closely associated with the rate of disease progression. In the early stages, characterized by regular astigmatism, satisfactory results can be achieved with the use of eyeglasses. In the advanced stages, a wide variety of CL options can be used for irregular astigmatism.^16^

In the multicenter and long-term CLEK (Collaborative Longitudinal Evaluation of KC) study, the main results of which were published in 1998, it was reported that only 16% of patients diagnosed with KC used spectacles as a primary optical device, 65-75% used CLs, mostly rigid gas-permeable CLs (RGPCLs), and surgical methods were preferred to treat the 10-20% of patients for whom these methods were not effective.^9^ In their study reviewing treatment methods used in the past 20 years, Mandathara et al.^17^ reported that CLs remain the main method for the treatment of KC, and that their use is associated with only reversible complications that do not threaten vision. They also stated that surgical methods such as intracorneal ring segment or phakic intraocular lens implantation can be used to treat patients who are unable to use CLs and that lamellar or full-thickness corneal transplantation may become necessary in case of extreme corneal thinning or scarring, but these surgical interventions can often result in temporary inflammatory responses and permanent sequelae.^17^

CL fitting is a long and arduous process for both the patient and the practitioner, and the three main goals of this process are to achieve optimal comfort, increase vision quality, and provide the best possible fit for the irregular corneal structure. Today, a wide array of lens options are used to achieve these goals, including corneal, semi-scleral, and scleral lenses, which differ in diameter, and rigid, soft, and hybrid lenses or piggyback lens systems, which differ in the materials used.^9,13^

In this review, we discuss these CLs in terms of visual performance, fitting characteristics, options for changing and combined use to increase success, and potential complications, in light of technological advances and current scientific studies.

## Rigid Gas-Permeable Contact Lenses in Keratoconus

RGPCLs are the type most frequently in KC, have the highest level of optical success, and therefore also increase the likelihood of nonsurgical management.^9,10^ In a study including 518 KC patients, Bilgin et al.^18^ reported that RGPCLs delayed the need for surgery in 98.9% of the patients.

The main reasons for the optical success of RGPCLs are that they form a smooth, spherical anterior optical surface that provides the main refractive effect, as well as shape the tear layer between the CL and the cornea into a liquid lens, thereby masking anterior surface irregularities that arise due to the ectatic cornea and the increased higher-order aberrations associated with these irregularities.^19^ However, Negishi et al.^20^ noted in a visual performance study based on contrast sensitivity measurement that even though keratoconic eyes had improved corrected visual acuity with RGPCLs, their visual performance was still lower than normal eyes with and without RGPCLs. The authors stated that this low visual performance may be attributable to the remaining higher-order aberrations in the keratoconic eye with RGPCL.

In addition, it is difficult to achieve optimal comfort with RGPCLs due to their rigidity. For this reason, patient motivation plays a key role in the use of CLs. Informing patients realistically after diagnosis about the fact that KC is a progressive, lifelong disease and about the relative advantages of CLs compared to other invasive treatment options is important for patients’ psychological preparedness.^13,14^

**Figure 1:**
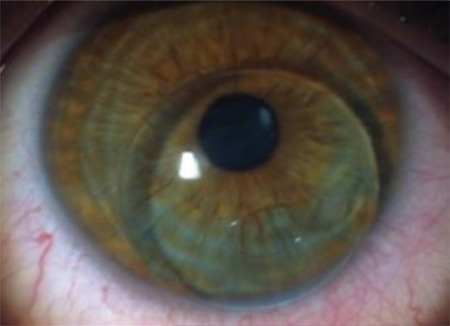
Displacement and binding of a gas-permeable rigid contact lens that shifted from the superior corneal region to the inferior quadrant where the cone is located

**Figure 2:**
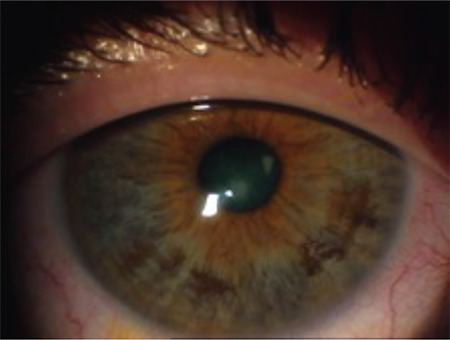
Displacement and binding of a gas-permeable rigid contact lens that shifted from the superior corneal region to the inferior quadrant where the cone is located

Another issue in CL fitting in KC is that the keratoconic topography poses technical problems regarding lens stabilization. In the corneal topography of KC, the steep ectatic area is usually displaced toward the inferior, inferonasal, or inferotemporal direction. The cone may be round and near-center or oval-shaped and sagging. If a diagram was made of the morphological changes that take place, the superior paralimbal surface would be flatter and larger, while the inferior paralimbal surface would be steeper and shorter. This topographic structure causes displacement of the RGPCL from the superior corneal region to the inferior quadrant where the cone is located, resulting in lens binding (Figure 1). With centered cones, sustained contact with a rigid CL can lead to epithelial trauma, erosions, and hypertrophic scarring at the cone apex (Figure 2). These are all important points to consider in the application of CLs.^13,21^

To overcome these issues, RGPCL designs with different diameter and base curve (BC) parameters have been produced. Corneal RGPCLs have a diameter of 8-10 mm and are more effective for central and mild cones. There are also intralimbal lenses with diameters of 10.5-12 mm.^21^ The options for BC, which is another important parameter, can range from standard single curve, double curve, and multi-curve lenses to reverse-geometry lenses.^22,23,24^

**Figure 3:**
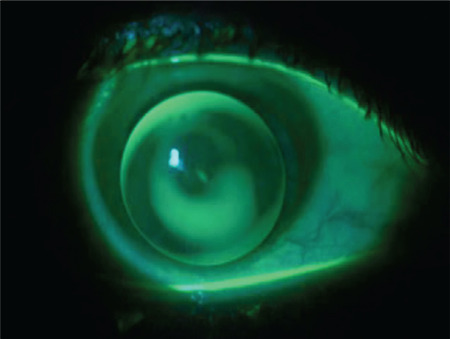
Fluorescein pattern of a multi-curve lens: minimal fluorescein in the center, pooling in the paracentral area, and a fluorescein-free area in the periphery

While spherical and aspheric designs can be used in early KC, successful outcomes can be obtained in advanced KC with multicurve posterior surface designs that have been developed to be compatible with the highly altered corneal topography and provide better stabilization. Among the multicurve RGPCLs, the outcomes of clinical application of Rose K2 lenses have been investigated in numerous studies. These lenses have a small optic zone diameter and 5-6 spherical curves on the back surface joined by gentle transitions, similar to an aspheric design (Figure 3).^25,26^ In addition, other multicurve lenses in which the spherical posterior optical zones are joined and designs produced using personalized curve parameters are also multicurve lens options with reports evincing their successful clinical application.^17,23^

Special CL fitting techniques have been developed in order to minimize contact with the cornea and prevent inferior displacement during wear. Approaches to RGPCL fitting for KC patients include apical bearing, apical clearance/peripheral bearing, and three-point touch.^21^

The apical bearing approach uses rigid CLs with large diameter and flat BC that rest on the corneal apex. However, this approach may cause epithelial erosion and, in later stages, superficial hypertrophic scarring due to apical pressure. In the apical clearance approach, the goal is to completely eliminate contact between the corneal apex and the rigid lens. Lenses with a small diameter and steep BC are used for this purpose. The most common problems encountered with this approach are mechanical and hypoxic corneal interactions due to the lens landing on the peripheral cornea and binding.^21,23,27,28^

**Figure 4:**
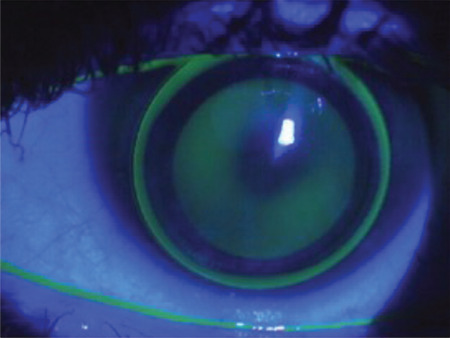
The three-point-touch approach: the lens lightly touches the corneal apex while bearing mostly on two separate points in the mid-periphery at 180 degrees from the apex

The goal of the three-point-touch approach is to ensure that the lens lightly touches the corneal apex while also being supported at two points in the mid-periphery at 180 degrees from the apex (Figure 4). With this method, the weight of the lens is largely distributed to healthy areas of the cornea with maximum protection of the apex.^13,23,27,28^ Although other methods are used when necessary, three-point-touch is the most popular approach in contemporary practice.

Several studies have shown that adverse effects at the cellular and molecular level may occur on the ocular surface and tear film layer of patients who use soft or rigid CLs compared to those who do not use CLs.^29,30^ In their study of the effects of rigid and soft lenses on the ocular surface, Pisella et al.^30^ demonstrated the presence of subclinical inflammation in asymptomatic CL users, although at a lower level in soft CL users than in rigid CL users.

Moon et al.^31^ compared myopic patients and KC patients using RGPCLs with KC patients not using CLs and a normal control group in terms of ocular surface and tear film changes. When the study data were evaluated, it was found that tear film break-up time, goblet cell count, and epithelial cell morphology had changed significantly in both groups using CLs compared to those not using CLs. The authors reported that the changes in CL users may be directly associated with CL use rather than keratoconic morphology.

On the other hand, it was determined that interleukin-6, tumor necrosis factor -alpha, ICAM-1, and VCAM-1 levels were elevated in the tears of KC patients using RGPCLs and that this increase was even greater in patients with advanced KC.^32^ Similar studies on KC patients reported an increase in lacrimal proinflammatory cytokine expression, and noted that changes in the concentration and dynamics of these mediators may influence the progression of the disease.^33,34^ Bitirgen et al.^35^ detected a decrease in basal epithelial cells and anterior stromal keratocyte density in corneas affected by KC, but reported no change in posterior stromal keratocyte density, endothelial cell count, or subbasal corneal nerve morphology. Bozkurt et al.^36^ used corneal topography and noncontact endothelial microscopic data to investigate changes in endothelial density in different stages of KC and determined that endothelial cell count decreased with KC progression.

In another study based on confocal microscopy, Erie et al.^37^ detected a decrease in keratocyte density in patients using RGPCLs compared to those using soft toric lenses and suggested that this may be due to apoptotic cell death and increased cytokine release associated with epithelial damage.

Although changes in corneal curvature due to CL use can occur with all lenses, they are more common in rigid CLs users and can even lead to clinical manifestations that mimic KC (corneal warpage syndrome). These changes increase in relation to CL material properties, design, fitting technique, and daily duration of use. Since the daily duration of use will be longer for KC patients, the risk is higher and may cause permanent changes in the cornea.^38^ However, Hwang et al.^39^ concluded based on the results of their study that successfully fitted multicurve RGPCLs do not have a morphological effect on the progression of KC.

Today, RGPCLs remain the first-line treatment for KC because they are practical and safe lenses with high optical success when applied in consideration of the many facets of KC.

## Soft Contact Lenses in Keratoconus

Despite the optical success of RGPCLs, lens intolerance resulting from irritation to the eyelid and anterior surface of the cornea due to their rigidity necessitates the use of soft lenses by some patients. However, soft contact lenses (SCLs) transfer corneal anterior surface irregularities to their own front surfaces and thus have low visual success rates. In the early disease stages, spherical SCLs and toric SCLs may be effective though myopia and astigmatism correction, but the same success is not seen in more advanced stages.^40^

In a study comparing visual success and ocular aberrations in KC patients using toric SCLs, RGPCLs, and spectacles, 22 patients with KC (16 wearing RGPCL and 6 wearing eyeglasses) were fitted with toric SCLs. It was reported that RGPCLs provided significantly better low-contrast visual acuity than toric SCLs, and that high-contrast visual acuity was also better with RGPCLs but the difference was not statistically significant. In the group of patients using spectacles, toric SCLs provided similar visual success to spectacles but were more successful at reducing higher-order aberrations with the exception of spherical aberrations.^40^

Recently developed special soft toric lenses feature increased central thickness for enhanced masking effect, toric front surface and aspherical surfaces for aberration reduction, and because oxygen permeability decreases with increasing thickness, a silicone hydrogel composition and thinner peripheral zone for improved oxygen supply and comfort. Some of these soft CLs designed specifically for KC include the HydroCone^®^ (Toris K) (SwissLens, Prilly, Switzerland) and KeraSoft^®^ IC (Bausch & Lomb Inc., Rochester, NY).

**Figure 5:**
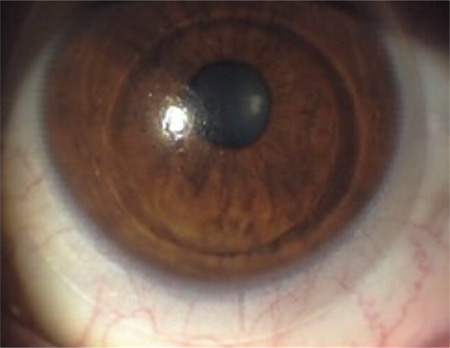
Toris K lens fitting

Toris K lenses have a toric front surface and provide dynamic stabilization. They are made from silicone hydrogel (Definitive 74%, Igel 77%) and come in two different central thickness options which are chosen according to KC severity. Toris K 12 (central thickness: 0.45 mm) can be used by patients with grade 1-2 KC, while Toris K 34 (central thickness: 0.52 mm) can be used by patients with grade 3-4 KC (Figure 5).

KeraSoft IC lenses are prism-ballasted, silicone hydrogel (Folcon V3, 74% water content) lenses with an aspheric toric front surface and aberration control. The lens periphery can be modified independently of the BC, and in addition to the standard peripheral curve option, it is also possible to change the customized quadrant design using the sector management control system. This enables the use of these CLs on many different cornea shapes and provides increased vision quality.

A study investigating the visual success and reliability of Toris K lenses of 50 keratoconic patients (64 eyes), Sultan et al.^41^ reported that best corrected visual acuity (BCVA) was significantly better with Toris K lenses compared to spectacles but did not differ statistically between RGPCLs and Toris K lenses. They concluded that Toris K lenses are a good alternative for keratoconic patients who are unable to tolerate RGPCLs. Gumus and Karaman et al.^42^ reported in their study of the visual success and comfort of Toris K lenses that the use of these lenses resulted in a mean BCVA increase of 4.5 lines, significantly better point spread function values, and high comfort ratings.

Yildiz et al.^43^ compared the effects of RGPCLs and silicone hydrogel KC lenses (Kerasoft IC and Toris K) on quality of life in keratoconic patients and found that both lens groups had similar quality of life scores determined using the CL Impact on Quality of Life questionnaire.

These soft toric lens designs with customized thickness and peripheral features are also reported to be a comfortable option that can improve vision quality in patients with trauma-induced irregular astigmatism and ectasias, as well as keratoconic patients with intracorneal ring segment implants.^44,45,46^

Meanwhile, customized SCLs with controlled optical power profiles are one of the current research topics in the area of CL applications in KC. Studies on this topic have gained momentum in recent years due to our increased knowledge on the nature of the optical deviations in KC and the technological capacity to produce alternatives for this.

Many studies have demonstrated that keratoconic patients have substantially more higher-order aberrations such as vertical coma arising from the anterior and posterior corneal surfaces due to asymmetry between the superior and inferior quadrants, highlighting the role of vertical coma values in addition to pachymetric and topometric indices in KC diagnosis and treatment planning.^47,48,49,50^ In a recent study conducted by Jinabhai et al.^51^ with the aim of achieving higher vision quality in keratoconic patients by correcting comatic optical aberrations, the authors stated that although customized CLs produced using a wavefront analysis system resulted in significant reduction of optical aberrations, visual performance was below the expected level, and that lens decentration may have a major role in this.

Aiming to lower the production cost of CLs with controlled optical power profiles, Suzaki et al.^52^ conducted trials with CLs produced with correction curves of standardized amount and angle of coma aberrations that are similar for most keratoconic patients, as an alternative to completely customized CLs. They trialed these lenses in patients with mild or suspected KC who were using RGPCLs and had less than perfect vision quality due to aberrations arising from the back surface, and found that they were able to reduce vertical coma aberrations in some of the patients and to increase visual acuity when used together with spectacles.

In conclusion, although there has been important progress toward combining comfortable wear and visual performance with soft KC lenses, it seems that this goal has not been entirely achieved and that the search in this field will continue.

## The Piggyback Contact Lens System in Keratoconus

**Figure 6:**
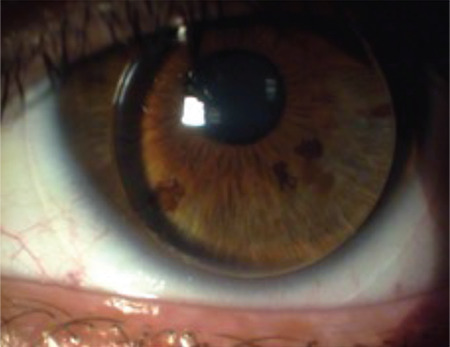
Piggyback contact lens application: a soft lens beneath a compatible rigid gas-permeable contact lens

The piggyback or dual lens system is the practice of placing a RGPCL on top of a soft “bandage” lens with high oxygen permeability to combine the comfortable wear of a soft lens with the optical performance of a rigid lens (Figure 6).

The piggyback CL (PBCL) system was first introduced in 1970 as a solution for keratoconic patients who were unable to use rigid lenses, but had limited success due to the low oxygen permeability of the lens materials used.^53^ Today, PBCL systems made with a combination of high-Dk silicone hydrogel and gas-permeable rigid materials have been shown to allow adequate oxygen to reach the cornea due to the high oxygen-permeability of both lenses. In addition, as the movement of both lenses promotes circulation of the tear layer between the lenses in this system, it is possible to benefit from the oxygen dissolved in the tears.^54^

The PBCL system may be preferable for keratoconic patients who experience discomfort and intolerance, inadequate lens stabilization, or apical epithelial erosion with RGPCLs.^55,56^ There are also reports of this system providing optimal CL fitting for patients with residual or progressing corneal irregularities after surgical procedures such as intracorneal ring segment implantation or cornea transplantation.^57,58^

**Figure 7:**
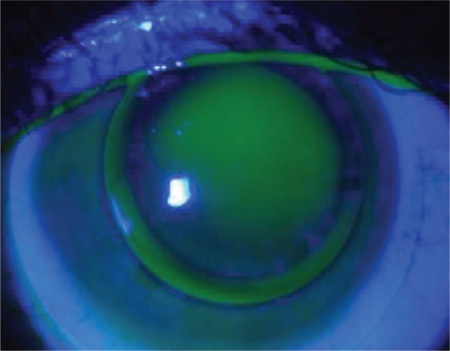
Fluorescein pattern of piggyback contact lens fitting: slight pooling at the center, a fluorescein-free zone in the periphery, and thin fluorescein accumulation at the lens edge

The goal in an optimal PBCL fitting is for the soft and rigid CLs to move independently but consistently with one another. After the soft lens is fitted and its movement on the eye surface is assessed, keratometric measurements are obtained from the front surface of this lens and the BC of the rigid lens is determined accordingly. After fitting the rigid lens, fluorescein is used to test the compatibility of the lenses with the eye and one another (Figure 7). When applying the PBCL system, many researchers prefer **positive-powered (+0.50 to +4.0)** soft CLs due to their steeper front surface curves for better stabilization of the rigid lens.^55,56,59^

On the other hand, a study by Sengor et al.^55^ showed that a large majority of patients were able to wear their rigid lenses without a soft lens after a mean of 6 months (3-12 months), which was attributed to reduced sensitivity and habituation over time.

In summary, with the bandage effect provided by the soft lens, the PBCL system is currently a successful and reliable method that can be used in KC patients to protect the corneal surface from mechanical effects, provide better stabilization of the RGPCL on the irregular cornea, and improve CL tolerance.

## Hybrid Contact Lenses in Keratoconus

Hybrid CLs (HCLs) are produced by fusing parts made of two different materials, rigid at the center and soft at the periphery, using a special technology. This type of CL aims to combine the visual performance of rigid lenses with the comfortable wear of soft lenses.^60^

Saturn II (OPSM, Contact Lenses, USA) and SoftPerm (Sola/Barnes-Hind Incorporated), the first hybrid lenses produced, led to problems associated with corneal hypoxia due to their low oxygen permeability, lens damage due to structural instability (particularly tears along the fusion line), and subsequent financial losses.^61^

Today, these problems have largely been overcome by using materials with high oxygen permeability. Of these, SynergEyes KC (SynergEyes Inc., Carlsbad, CA) HCLs were produced considering the KC BC using a rigid, high-Dk material at the center, hydrogel material for the periphery, and a reinforced fusion zone. They were immediately followed by the introduction of another HCL, ClearKone lenses. The rigid part of ClearKone lenses is made of Paragon HDS 100 (Paragon Vision Sciences, Mesa, AZ) gas-permeable rigid material, with a dome (vault) diameter of 7.4 mm and oxygen permeability of 100x10^-11^ (cm^2^/s) x (mLO_2_/[mL x mmHg]). The rigid center part has a spherical optical zone and a reverse-geometry curve. The soft skirt section is made of nonionic hydrogel material with 27% water content and an oxygen permeability of 9.3x10^-11^ (cm^2^/second) x (mLO_2_/[mL x mmHg]), and can be up to 14.5 mm in diameter.

**Figure 8:**
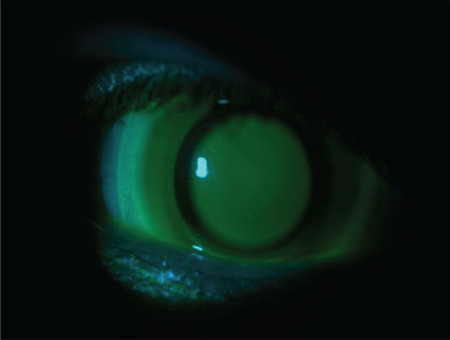
Hybrid contact lens fitting: no contact in the center, minimal contact along the rigid-soft junction

When fitting these lenses, the rigid and soft parts should each be considered individually; separate measurements are required to ensure the central dome height (vault) of the rigid part provides minimal clearance of the cone while the skirt curvature of the soft part provides adequate support. Optimal fitting aims for complete central clearance (with no air bubbles) and a soft landing at the lens fusion area (Figure 8). In this design, a steeper skirt increases lens movement and prevents lens adhesion. As an optical feature, they cannot correct lenticular astigmatism but provide good results in irregular corneas without residual astigmatism. To prevent hypoxia and vascularization that may be caused by a hydrogel skirt with low Dk, SynergEyes UltraHealth lenses were produced by changing the skirt material to silicone hydrogel.^60,62^

Although studies on hybrid lenses are limited, it was reported in a study on SynergEyes KC, a new-generation hybrid lens, that successful results were obtained in 87% of 61 eyes with KC (58 subjects) and pellucid marginal degeneration (PMD) (3 subjects).^63^ In another study evaluating the clinical data and quality of life scores of keratoconic patients wearing ClearKone lenses and RGPCLs, it was reported that although both lens types provided similar visual quality, vision-related quality of life scores were higher with the ClearKone SynergEyes SCLs.^64^

However, Fernandez-Velazquez^65^ pointed out complications that may arise during the use of ClearKone SCLs, and stressed that early circular corneal clouding may be a harbinger of a serious problem and that SCL use should be discontinued in such cases.

In conclusion, although HCLs are a product of advanced technology that combines the positive qualities of rigid and soft materials in a single lens, more research is needed on the effects they may have on the cornea and ocular surface in the long term.

## Scleral Lenses in Keratoconus

Scleral lenses are 15 mm or larger in diameter and rest on the sclera. Mini-scleral lenses are 15-18 mm in diameter, whereas large scleral lenses are over 18 mm in diameter and have a zone of 6 mm or larger resting on the sclera.^21^ In 2019, the Scleral Lens Education Society adopted a new perspective and defined scleral lenses as “a lens fitted to vault over the entire cornea, including the limbus, and to land on conjunctiva overlying the sclera.” Thus, the use of the term scleral lens was accepted for all lenses that rest on the sclera, removing the mini-/large scleral lens distinction.^66^

**Figure 9:**
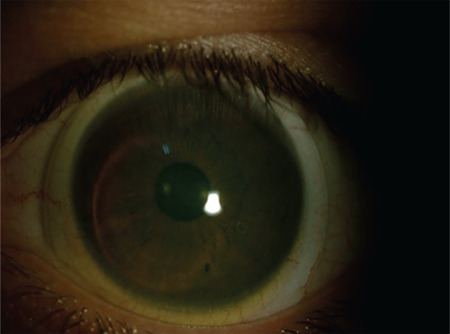
Scleral lens fitting: light contact with the sclera, no signs of pressure

Scleral lenses rest on the sclera and do not contact the cornea because the lens vault creates a space between the lens and the cornea, called corneal clearance. Before applying to the eye, the lens is filled with saline (0.9% sodium chloride) solution (Figure 9). This fluid reservoir between the lens and the cornea supports the scleral lens, prevents corneal desiccation, and optically neutralizes aberrations caused by corneal surface irregularities.^66,67^

Scleral lenses are most commonly used to treat patients with KC that causes high irregular astigmatism. Gas-permeable scleral lenses help reduce the need for keratoplasty in KC patients by providing a safe and successful treatment alternative in terms of visual acuity and comfort in cases where other CL options have been unsuccessful.^68,69,70^ Other indications include ectatic diseases of the cornea such as PMD, keratoglobus, and post-keratoplasty astigmatism, as well as ocular surface diseases such as Stevens-Johnson syndrome, dry eye, graft-versus-host disease, and ocular cicatricial pemphigoid due to its liquid bandage effect.^67,71,72^

Since scleral lenses have no contact with the sensitive cornea, lens awareness and mechanical stress on the cornea are very low compared to other lens types. The fluid reservoir between the lens and the ocular surface contributes to the moistening of the ocular surface, providing an advantage in terms of dry eye associated with CL use.^72^ Bergmanson et al.^73^ observed that scleral lenses were more advantageous for KC patients in terms of comfort and vision and thus were more preferred than soft, piggyback, hybrid, and rigid gas-permeable lenses. On the other hand, although scleral lenses alleviate the feeling of dryness, more than half of patients using these lenses were reported to have blurred vision during the day.

In scleral lens applications, choice of total lens diameter is important for a successful fitting. Modern scleral lenses are produced with smaller diameters compared to the original lenses. It is recommended to start fitting with the smallest possible diameter that is compatible with the landing zone diameter, horizontal visible iris diameter (HVID), and limbus width, and transition to larger diameter lenses as necessary.

Mini-scleral lenses can be made thinner, which provides an advantage in terms of oxygen permeability. Reduced lens mass and less movement than larger lenses make them easier to tolerate.^74^ Mini-scleral lenses have lower corneal clearance compared to large scleral lenses because with smaller lens diameter, the sagittal depth necessary for the lens to clear the corneal apex decreases. It is recommended that corneal clearance should ideally be between 100 and 300 µm in the central zone. Low central clearance results in increased oxygen transmission, better visual acuity, and less bubble formation. Since limbal clearance is also lower with mini-scleral lenses, less debris gets under the lens and there is less mechanical stress on the limbus and weight-induced downward decentration of the lens. In addition, mini-scleral lenses are less affected by scleral toricity.^71,75^ That being said, small-diameter lenses fit more tightly and may adhere to the cornea due to a vacuum effect, and their removal is more difficult compared to large-diameter lenses. To reduce the vacuum effect, air ventilation can be provided via perforations on the lens or grooves in the back surface of the lens.^76^

Large scleral lenses, on the other hand, are preferred for steep cones that require more sagittal depth, PMD, keratoglobus, and ocular surface diseases. With large scleral lenses, the vault is higher, the weight of the lens is better distributed across the sclera, and there is less lens-lid interaction. However, these lenses also have higher mass and are more affected by gravity. There may be decentration of the lens due to the lid effect, and they are more difficult to apply. Special designs and a toric back surface in large scleral lenses may help prevent sectoral pressure, bubble formation, tear exchange, conjunctival prolapse, and lens decentration and distortion.^74^

Scleral lenses consist of 3 zones: The scleral (haptic) zone that rests on the sclera, the dome (vault) that provides corneal and limbal clearance, and the optical zone. The optical zone is usually 0.2 mm larger than the HVID. In scleral lens fitting, the ocular performance of the lens is affected by the haptic zone, corneal and limbal clearance, and the lens edge. Scleral lens fitting is different from rigid lens fitting. The fitted lens does not move on the eye, and the most important point to consider is compatibility of the haptic and sclera. Depending on the toricity of the sclera, there may be edge lift in some patients. In such cases, a toric structure can be added to scleral lenses to reduce accumulation of debris between the cornea and lens, thereby enabling prolonged use and improved comfort.^77^

While corneal topography is of great importance with rigid corneal lenses, it is not believed to be helpful in scleral lens fitting.^78^ In their study of patients fitted with mini-scleral (15 mm) lenses, based on cone center location and size index values obtained with corneal topography, Fernández-Velázquez et al.^79^ reported that corneal apex curvature values (diopters) were positively correlated with the average sagittal depth of the last fitted lens. In the same study, final lens selection was based on the compatibility between the cornea and the lens back surface as determined by anterior segment OCT.

Anterior segment OCT imaging is currently used during scleral lens fitting for initial lens selection, assessment of lens compatibility with the cornea and sclera, and evaluation the ocular responses in lens users. It can also provide useful information regarding scleral thickness, curvature, and toricity in addition to a detailed evaluation of the corneoscleral-limbal anatomy.^80^

With scleral lenses, greater lens thickness and the fluid layer behind the lens may lead to signs of corneal hypoxia. It is believed that the high-Dk materials and thin designs of modern scleral lenses will eliminate this hypoxic effect in the cornea, but there is no evidence demonstrating this definitively. Although a scleral lens with a Dk of 100 will theoretically have adequate oxygen permeability, the Dk of tears is only 80; therefore, the thickness of the tear reservoir behind the scleral lens may reduce its oxygen permeability.^81^ Pullum and Stapleton^82^ demonstrated that after 3 hours of wearing a scleral lens 0.6 mm thick with a Dk value of 115 Barrer, the resulting corneal edema was below the physiological limit of 3%. In order to prevent symptoms of corneal hypoxia and edema in scleral lens wearers, it is recommended to use scleral lenses up to 200 µm thick and made from high-Dk material (>125 Barrer) fitted with a clearance less than 150 µm.^83^

Scleral lenses have improved significantly in recent years in terms of their material properties, design, and production methods.^79^ PROSE, formerly known as Boston scleral lens, Boston scleral lens prosthetic device, or Boston ocular surface prosthesis, is a customized lens with computer-assisted design. With spline technology, the lens haptic fits the sclera better, indentation is minimal, channels can be added to the lens when desired, and due to its front surface eccentricity, visual acuity can also be improved in patients with advanced keratoconus.^84^ In keratoconic patients, a wavefront-correction-based optical feature can be added to scleral lenses to reduce higher-order aberrations and improve vision.^85^ Front surface eccentricity and asphericity can also increase vision level in patients with advanced KC by ensuring lens flattening from the center toward the periphery.^86,87^ Today, multifocal scleral lenses are also produced for presbyopic patients with keratoconus.^88^

With their expanding areas of usage and technological advances, modern scleral lenses are candidates to become a successful option for contemporary CL fitting in KC, especially advanced cases.

In conclusion, CL application in KC continues to have a primary role in the elimination of optical problems associated with this disease. To date, various types of CL have been developed and span a wide range, including rigid lenses, soft lenses, lenses that combine the positive qualities of both of these materials, and lenses with custom designs. Therefore, the likelihood of being able to find a solution to the problems faced by KC patients has increased substantially and it has become possible to offer patients these reversible and highly varied options before resorting to surgery. All of these recent improvements could be an important opportunity for a patient who is sufficiently informed about living with their chronic illness and not losing their chance for surgery in the future.

## References

[1] Aksoy S, Akkaya S, Özkurt Y, Kurna S, Açıkalın B, Şengör T (2017). Topography and higher order corneal aberrations of the fellow eye in unilateral keratoconus. Turk J Ophthalmol..

[2] Millodot M, Ortenberg I, Lahav-Yacouel K, Behrman S (2016). Effect of ageing on keratoconic corneas. J Optom..

[3] Tuncer Z, Soylu T (2012). Use of Contact Lenses in Eyes with Severe Keratoconus: Long-term Results. Turk J Ophthalmol..

[4] Jonas JB, Nangia V, Matin A, Kulkarni M, Bhojwani K (2009). Prevalence and associations of keratoconus in rural Maharashtra in Central India: the central India eye and medical study. Am J Ophthalmol..

[5] Kennedy RH, Bourne WM, Dyer JA (1986). A 48-year clinical and epidemiologic study of keratoconus. Am J Ophthalmol..

[6] Godefrooij DA, de Wit GA, Uiterwaal CS, Imhof SM, Wisse RP (2017). Age-specific incidence and prevalence of keratoconus: a nationwide registration study. Am J Ophthalmol..

[7] Woodward MA, Blachley TS, Stein JD (2016). The Association between sociodemographic factors, common systemic diseases, and keratoconus: an analysis of a nationwide heath care claims database. Ophthalmology..

[8] Karolak JA, Gajecka M (2017). Genomic strategies to understand causes of keratoconus. Mol Genet Genomics..

[9] Zadnik K, Barr JT, Edrington TB, Everett DF, Jameson M, McMahon TT, Shin JA, Sterling JL, Wagner H, Gordon MO (1998). Baseline findings in the Collaborative Longitudinal Evaluation of Keratoconus (CLEK) study. Invest Ophthalmol Vis Sci..

[10] Gomes JA, Tan D, Rapuano CJ, Belin MW, Ambrósio Jr R, Guell JL, Malecaze F, Nishida K, Sangwan VS;, Group of Panelists for the Global Delphi Panel of Keratoconus and Ectatic Diseases (2015). Global consensus on keratoconus and ectatic diseases. Cornea.

[11] Atilano SR, Lee DH, Fukuhara PS, Chwa M, Nesburn AB, Udar N, Cristina Kenney M (2019). Corneal Oxidative Damage in Keratoconus Cells due to Decreased Oxidant Elimination from Modified Expression Levels of SOD Enzymes, PRDX6, SCARA3, CPSF3, and FOXM1. J Ophthalmic Vis Res..

[12] Gokul A, Vellara HR, Patel DV (2018). Advanced anterior segment imaging in keratoconus: a review. Clin Exp Ophthalmol..

[13] Wagner H, Barr JT, Zadnik K (2007). Collaborative Longitudinal Evaluation of Keratoconus (CLEK) Study: methods and findings to date. Cont Lens Anterior Eye..

[14] Kymes SM, Walline JJ, Zadnik K, et al;, Collaborative longitudinal evaluation of keratoconus study group (2004). Quality of life in keratoconus. Am J Ophthalmol.

[15] Aydin Kurna S, Altun A, Gencaga T, Akkaya S, Sengor T (2014). Vision related quality of life in patients with keratoconus. J Ophthalmol..

[16] Rico-Del-Viejo L, Garcia-Montero M, Hernández-Verdejo JL, García-Lázaro S, Gómez-Sanz FJ, Lorente-Velázquez A (2017). Nonsurgical Procedures for Keratoconus Management. J Ophthalmol..

[17] Mandathara PS, Stapleton FJ, Willcox MDP (2017). “Outcome of keratoconus management: review of the past 20 years’ contemporary treatment modalities”. Eye Contact Lens..

[18] Bilgin LK, Yilmaz S, Araz B, Yuksel SB, Sezen T (2009). 30 years of contact lens prescribing for keratoconic patients in Turkey. Cont Lens Anterior Eye..

[19] Dorronsoro C, Barbero S, Llorente L, Marcos S (2003). On-Eye Measurement of Optical Performance of Rigid Gas Permeable Contact Lenses Based on Ocular and Corneal Aberrometry. Optom Vis Sci..

[20] Negishi K, Kumanomıdo T, Utsumı Y, Tsubota K (2007). Effect of Higher-Order Aberrations on Visual Function in Keratoconic Eyes with a Rigid Gas Permeable Contact Lens. Am J Ophthalmol..

[21] Barnett M, Mannis MJ (2011). Contact lenses in the management of keratoconus. Cornea..

[22] Lunardi LH, Arroyo D, Andrade Sobrinho MV, Lipener C, Rosa JM (2016). Descriptive analysis of the type and design of contact lenses fitted according to keratoconus severity and morphology. Arq Bras Oftalmol..

[23] Hwang JS, Lee JH, Wee WR, Kim MK (2010). Effects of multicurve RGP contact lens use on topographic changes in keratoconus. Korean J Ophthalmol..

[24] Hu CY, Tung HC (2008). Managing keratoconus with reverse-geometry and dual-geometry contact lenses: a case report. Eye Contact Lens..

[25] Betts AM, Mitchell GL, Zadnik K (2002). Visual performance and comfort with the Rose K lens for keratoconus. Optom Vis Sci..

[26] Ozkurt YB, Sengor T, Kurna S, Evciman T, Acikgoz S, Haboğlu M, Aki S (2008). Rose K contact lens fitting for keratoconus. Int Ophthalmol..

[27] Leung KKY (1999). RGP fitting philosophies for keratoconus. Clin Exp Optom..

[28] Downie LE, Lindsay RG (2015). Contact lens management of keratoconus. Clin Exp Optom..

[29] Sengor T, Kurna SA, Ozbay N, Ertek S, Aki S, Altun A (2012). Contact lens-related dry eye and ocular surface changes with mapping technique in long-term soft silicone hydrogel contact lens wearers. Eur J Ophthalmol..

[30] Pisella PJ, Malet F, Lejeune S, Brignole F, Debbasch C, Bara J, Rat P, Colin J, Baudouin C (2001). Ocular surface changes induced by contact lens wear. Cornea..

[31] Moon JW, Shin KC, Lee HJ, Wee WR, Lee JH, Kim MK (2006). The effect of contact lens wear on the ocular surface changes in keratoconus. Eye Contact Lens..

[32] Lema I, Duran JA, Ruiz C, Diez-Feijoo E, Acera A, Merayo J (2008). Inflammatory response to contact lenses in patients with keratoconus compared with myopic subjects. Cornea..

[33] Fodor M, Kolozsvári BL, Petrovski G, Kettesy BA, Gogolák P, Rajnavölgyi E, Ujhelyi B, Módis L, Petrovski BE, Szima GZ, Berta A, Facskó A (2013). Effect of contact lens wear on the release of tear mediators in keratoconus. Eye Contact Lens..

[34] Wisse RP, Kuiper JJ, Gans R, Imhof S, Radstake TR, Van der Lelij A (2015). Ocul Surf. Cytokine Expression in Keratoconus and its Corneal Microenvironment: A Systematic Review..

[35] Bitirgen G, Ozkagnici A, Malik RA, Oltulu R (2013). Evaluation of contact lensinduced changes in keratoconic corneas using in vivo confocal microscopy. Invest Ophthalmol Vis Sci..

[36] Bozkurt B, Yılmaz M, Meşen A, Kamış Ü, Ekinci Köktekir B, Okudan S (2017). Correlation of Corneal Endothelial Cell Density with Corneal Tomographic Parameters in Eyes with Keratoconus. Turk J Ophthalmol.

[37] Erie JC, Patel SV, McLaren JW, Nau CB, Hodge DO, Bourne WM (2002). Keratocyte density in keratoconus. A confocal microscopy study. Am J Ophthalmol.

[38] Shokrollahzadeh F, Hashemi H, Jafarzadehpur E, Mirzajani A, Khabazkhoob M, Yekta A, Asgari S (2016). Corneal aberration changes after rigid gas permeable contact lens wear in keratokonic patients. J Curr Ophthalmol.

[39] Hwang JS, Lee JH, Wee WR, Kim MK (2010). Effects of multicurve RGP contact lens use on topographic changes in keratoconus. Korean J Ophthalmol..

[40] Jinabhai A, Radhakrishnan H, Tromans C, O’Donnell C (2012). Visual performance and optical quality with soft lenses in keratoconus patients”. Ophthalmic Physiol Opt..

[41] Sultan P, Dogan C, Iskeleli G (2016). A retrospective analysis of vision correction and safety in keratoconus patients wearing Toris K soft contact lenses. Int Ophthalmol..

[42] Gumus K, Kahraman N (2016). A new fitting approach for providing adequate comfort and visual performance in keratoconus: soft HydroCone (Toris K) lenses. Eye Contact Lens..

[43] Yildiz EH, Erdurmus M, Elibol ES, Acar B, Vural ET (2015). Contact lens impact on quality of life in keratoconus patients: rigid gas permeable versus soft silicone-hydrogel keratoconus lenses. Int J Ophthalmol..

[44] Altun A, Kurna SA, Sengor T, Altun G, Olcaysu O, Simsek MH (2015). Success of hydrocone (TORIS-K) soft contact lens for keratoconus and traumatic keratopathy. Pak J Med Sci..

[45] Carballo-Alvarez J, Puell MC, Cuina R, Diaz-Valle D, Vazquez JM, Benitez- Del-Castillo JM (2014). Soft contact lens fitting after intrastromal corneal ring segment implantation to treat keratoconus. Cont Lens Anterior Eye..

[46] Fernandez-Velazquez FJ, Fernandez-Fidalgo MJ (2015). Feasibility of custommade hydrogel contact lenses in keratoconus with previous implantation of intracorneal ring segments. Cont Lens Anterior Eye..

[47] Alió JL, Shabayek MH (2006). Corneal higher order aberrations: a method to grade keratoconus”. J Refract Surg..

[48] Kosaki R, Maeda N, Bessho K, Hori Y, Nishida K, Suzaki A, Hirohara Y, Mihashi T, Fujikado T, Tano Y (2007). Magnitude and orientation of Zernike terms in patients with keratoconus. Invest Ophthalmol Vis Sci.

[49] Hashemi H, Beiranvand A, Yekta A, Maleki A, Yazdani N, Khabazkhoobd M (2016). Pentacam top indices for diagnosing subclinical and definite keratoconus. J Curr Ophthalmol..

[50] Negishi K, Kumanomido T, Utsumi Y, Tsubota K (2007). Effect of higher-order aberrations on visual function in keratoconic eyes with a rigid gas permeable contact lens. Am J Ophthalmol..

[51] Jinabhai A, O’Donnell C, Tromans C, Radhakrishnan H (2014). Optical quality and visual performance with customised soft contact lenses for keratoconus. Ophthalmic Physiol Opt..

[52] Suzaki A, Maeda N, Fuchihata M, Koh S, Nishida K, Fujikado T (2017). Visual performance and optical quality of standardized asymmetric soft contact lenses in patients with keratoconus. Invest Ophthalmol Vis Sci..

[53] Polse KA, Decker MR, Sarver MD (1977). Soft and hard contact lenses worn in combination. Am J Optom Physiol Opt.

[54] Alemany Al, Meijome JMG, Almedia JB, Parafita MA, Refojo MF (2006). Oxygen transmissibility of piggyback systems with conventional soft and silicone hydrogel contact lenses. Cornea..

[55] Sengor T, Kurna SA, Akı S, Ozkurt Y (2011). High Dk piggyback contact lens system for contact lens-intolerant keratoconus patients. Clin Ophthalmol..

[56] O’Donnell C, Maldonado-Codina C (2004). A hyper-Dk piggyback contact lens system for keratoconus. Eye Contact Lens..

[57] Smith KA, Carrell JD (2008). High Dk piggybckcontact lenses over intacs for keratoconus: a case report. Eye Contact Lens.

[58] Wietharn BE, Driebe WT (2004). Fitting contact lenses for visual rehabilitation after penetrating keratoplasty. Eye Contact Lens..

[59] Andreanos KD, Hashemi K, Petrelli M, Droutsas K, Georgalas I, Kymionis GD (2017). Keratoconus Treatment Algorithm. Ophthalmol Ther.

[60] Nau AC (2008). A comparison of Synerg Eyes versus traditional rigid gas permeable lens designs for patients with irregular corneas. Eye Contact Lens.

[61] Ozkurt Y, Oral Y, Karaman A, Ozgur O, Dogan OK (2007). A retrospective case series: use of SoftPerm contact lenses in patients with keratoconus. Eye Contact Lens..

[62] Downie LE, Lindsay RG (2015). Contact lens management of keratoconus. Clin Exp Optom..

[63] Abdalla YF, Elsahn AF, Hammersmith KM, Cohen EJ (2010). Synerg Eyes lenses for keratoconus. Cornea..

[64] Hashemi H, Shaygan N, Asgari S, Rezvan F, Asgari S (2014). ClearKone-SynergEyes or rigid gas-permeable contact lens in keratoconic patients: a clinical decision. Eye Contact Lens..

[65] Fernandez-Velazquez FJ (2011). Severe epithelial edema in Clear-Kone SynergEyes contact lens wear for keratoconus. Eye Contact Lens..

[66] Michaud L, Lipson M, Kramer E, Walker M. The official guide to scleral lens terminology. Cont Lens Anterior Eye. 2019 Sep 24. pii: S1367-0484 (19)30219-X..

[67] Jacobs DS (2008). Update on scleral lenses. Curr Opin Ophthalmol.

[68] Koppen C, Kreps EO, Anthonissen L, Hoey MV, Dhubhghaill SN, Vermeulen L (2018). Scleral lenses reduce the need for corneal transplants in severe Keratoconus. Am J Ophthalmol..

[69] DeLoss KS, Fatteh NH, Hood CT (2014). Prosthetic Replacement of the Ocular Surface Ecosystem (PROSE) scleral device compared to keratoplasty for the treatment of corneal ectasia. Am J Ophthalmol..

[70] Segal O, Barkana Y, Hourovitz D, Behrman S, Kamun Y, Avni I, Zadok D (2003). Scleralcontact lenses may help where other modalities fail. Cornea..

[71] Pullum KW (2006). Scleral contact lenses: indications and current clinical methods. Optometry Today.

[72] Alipour F, Kheirkhah A, Jabarvand Behrouz M (2012). Use of mini scleral contact lenses in moderate to severe dry eye. Cont Lens Anterior Eye.

[73] Bergmanson JP, Walker MK, Johnson LA (2016). Assessing Scleral Contact Lens Satisfaction in a Keratoconus Population. Optom Vis Sci..

[74] Fadel D (2017). Modern scleral lenses: Mini versus large. Cont Lens Anterior Eye..

[75] Sclerals for normal corneas (2015). Beyond irregular: scleral lenses for everyday use, Cont. Lens Spectr..

[76] Rathi VM, Mandathara PS, Taneja M, Dumpati S, Sangwan VS (2015). Scleral lens for keratoconus: technology update. Clin Ophthalmol..

[77] Visser ES, Visser R, Van Lier HJ (2006). Advantages of toric scleral lenses. Optom Vis Sci..

[78] Schornack MM, Patel SV (2010). Relationship between corneal topographic indices and scleral lens base curve. Eye Contact Lens..

[79] Fernández-Velázquez FJ (2019). Performance and predictability of a new large diameter contact lens design in keratoconic corneae. Cont Lens Anterior Eye..

[80] Vincent SJ, Alonso-Caneiro D, Collins MJ (2019). Optical coherence tomography and scleral contact lenses: clinical and research applications. Clin Exp Optom..

[81] Bergamnson JPG, Ezekiel DF, van der Worp E (2015). Scleral contact lenses and Hypoxia. Theory versus practice, Cont. Lens Anterior Eye.

[82] Pullum KW, Stapleton FJ (1997). Scleral lens induced corneal swelling: what effect of varying Dk and lens thickness?. CLAO J.

[83] Compan V, Oliveira C, Aguilella-Arzo M, Mollá S, Peixoto-de-Matos SC, González Méijome JM (2014). Oxygen diffusion and edema with modern scleral rigid gas permeable contact lenses. Invest. Ophthalmol. Vis. Sci..

[84] Rosenthal P, Croteau A (2005). Fluid-ventilated,gas-permeablescleralcontact lens is an effective option for managing severe ocular surface disease and many corneal disorders that would otherwise require penetrating keratoplasty. Eye Contact Lens..

[85] Marsack JD, Ravikumar A, Nguyen C, Ticak A, Koenig DE, Elswick JD, Applegate RA (2014). Wavefront-guided scleral lens correction in keratoconus. Optometry Vision Sci..

[86] Gumus K, Gire A, Pflugfelder SC (2011). The impact of the Boston ocular surface prosthesis on wavefront higher-order aberrations. Am J Ophthalmol..

[87] Hussoin T, Le HG, Carrasquillo KG, Johns L, Rosenthal P, Jacobs DS (2012). The effect of optic asphericity on visual rehabilitation of corneal ectasia with a prosthetic device. Eye Contact Lens..

[88] Vincent SJ, Fadel D (2019). Optical considerations for scleral contact lenses: A review. Cont Lens Anterior Eye..

